# Reduction in the risk of human breast cancer by selective cyclooxygenase-2 (COX-2) inhibitors

**DOI:** 10.1186/1471-2407-6-27

**Published:** 2006-01-30

**Authors:** Randall E Harris, Joanne Beebe-Donk, Galal A Alshafie

**Affiliations:** 1 The Ohio State University College of Medicine and Public Health, 320 West 10^th ^Avenue, Columbus, Ohio, 43210-1240, USA

## Abstract

**Background:**

Epidemiologic and laboratory investigations suggest that nonsteroidal anti-inflammatory drugs (NSAIDs) have chemopreventive effects against breast cancer due to their activity against cyclooxygenase-2 (COX-2), the rate-limiting enzyme of the prostaglandin cascade.

**Methods:**

We conducted a case control study of breast cancer designed to compare effects of selective and non-selective COX-2 inhibitors. A total of 323 incident breast cancer patients were ascertained from the James Cancer Hospital, Columbus, Ohio, during 2003–2004 and compared with 649 cancer free controls matched to the cases at a 2:1 ratio on age, race, and county of residence. Data on the past and current use of prescription and over the counter medications and breast cancer risk factors were ascertained using a standardized risk factor questionnaire. Effects of COX-2 inhibiting agents were quantified by calculating odds ratios (OR) and 95% confidence intervals.

**Results:**

Results showed significant risk reductions for selective COX-2 inhibitors as a group (OR = 0.29, 95% CI = 0.14–0.59), regular aspirin (OR = 0.49, 95% CI = 0.26–0.94), and ibuprofen or naproxen (0.36, 95% CI = 0.18–0.72). Acetaminophen, a compound with negligible COX-2 activity and low dose aspirin (81 mg) produced no significant change in the risk of breast cancer.

**Conclusion:**

Selective COX-2 inhibitors (celecoxib and rofecoxib) were only recently approved for use in 1999, and rofecoxib (Vioxx) was withdrawn from the marketplace in 2004. Nevertheless, even in the short window of exposure to these compounds, the selective COX-2 inhibitors produced a significant (71%) reduction in the risk of breast cancer, underscoring their strong potential for breast cancer chemoprevention.

## Background

The recent recall of rofecoxib (Vioxx) from the marketplace due its alleged association with increased risk for cardiovascular disease has severely compromised further testing of all selective cyclooxygenase-2 (COX-2) inhibitors in the chemoprevention and therapy of cancer. Despite compelling evidence that COX-2 inhibitors have powerful anti-cancer effects, several clinical trials designed to evaluate these compounds in the chemoprevention and therapy of neoplasms have been discontinued or suspended [1,2].

Both the magnitude and the direction of effect of selective COX-2 blockers on the risk of cardiovascular disease is the subject of controversy. Risk *increases *have been observed with use of rofecoxib and celecoxib in clinical trials that were designed to evaluate their potential for treating arthritis or reducing colonic polyp recurrence [3-5], whereas risk *decreases *have been observed in observational studies that were designed to evaluate effects of these same compounds on cardiovascular diseases [6-8]. Still other investigations suggest that COX-2 inhibitors have no effect on the risk of myocardial infarction and related cardiovascular events [9,10].

Among American women, breast cancer is the most frequently diagnosed malignancy and second leading cause of cancer death [11]. Despite intensive efforts aimed primarily at early detection and therapy, the mortality rates of breast cancer have remained virtually constant for several decades. Innovative research efforts must therefore be redirected towards chemoprevention of the early stages of carcinogenesis. Among twenty published epidemiologic studies that focused on the association between intake of nonsteroidal anti-inflammatory drugs (NSAIDs) and the risk of human breast cancer, 13 reported statistically significant risk reductions. Meta-analysis of these data suggests that regular NSAID intake significantly reduces the risk of breast cancer [12].

Two selective COX-2 inhibitors, celecoxib (Celebrex) and rofecoxib (Vioxx), were approved for the treatment of arthritis by the United States Food and Drug Administration (FDA) in 1999. Until the recall of Vioxx in September, 2004, these two compounds plus other selective COX-2 inhibitors valdecoxib (Bextra) and meloxicam (Mobic) were widely utilized in the United States for pain relief and treatment of osteoarthritis and rheumatoid arthritis. The time period between approval of Celebrex to the recall of Vioxx provides an approximate six-year window for evaluation of exposure to such compounds by a case control approach. The current case control study was designed to test the chemopreventive value of selective COX-2 blockade against human breast cancer.

## Methods

We studied 323 cases of invasive breast cancer with histological verification based upon review of the pathology records, and 649 group-matched controls with no personal history of cancer and no current breast disease based on screening mammography. Cases were sequentially ascertained for interview at the time of their diagnosis during 2003 through September, 2004 at The Arthur G. James Cancer Hospital and Richard J. Solove Research Institute (CHRI), Columbus, Ohio. There were no refusals to participate among cases. The controls were ascertained from the mammography service of the cancer hospital during the same time period and frequency matched to the cases at a rate of 2:1 by five-year age interval, race, and place (county) of residence. Controls were sequentially ascertained for each matching category resulting in a stratified random sample. Among women eligible for participation, 95% completed the questionnaire.

Critical information on exposure to NSAIDs and other factors were obtained utilizing a standardized risk factor questionnaire. The questionnaires were administered in person by trained medical personnel prior to definitive surgery or treatment for the cases and at the time of screening mammography for controls. The data variables collected consisted of demographic characteristics, height, weight, menstrual and pregnancy history, family history of breast and ovarian cancer, comprehensive information on cigarette smoking, alcohol intake, pre-existing medical conditions (arthritis, chronic headache, cardiovascular conditions including hypertension, angina, ischemic attacks, stroke, and myocardial infarction, lung disease, and diabetes mellitus), and medication history including over the counter (OTC) and prescription NSAIDs, and exogenous hormones. Regarding selective COX-2 inhibitors and other NSAIDs, the use pattern (frequency, dose, and duration), and the type, (celecoxib, valdecoxib, rofecoxib, meloxicam, aspirin, ibuprofen, naproxen, indomethacin) were recorded. Data on the related analgesic, acetaminophen were collected for comparison with selective COX-2 inhibitors and other NSAIDs.

Case-control differences in means and frequencies were checked for statistical significance by t-tests and chi square tests, respectively. Effects of the selective COX-2 inhibitors as a group were quantified by estimating odds ratios and their 95% confidence intervals. Odds ratios were adjusted for age and classical breast cancer risk factors (parity, family history, body mass, menopausal status, chronic smoking, and regular alcohol intake) by logistic regression analysis [13,14]. Adjusted estimates were obtained for specific types of compounds, e.g., over the counter NSAIDs, selective COX-2 inhibitors, and acetaminophen.

## Results

Pertinent characteristics of the cases and controls are given in Table 1. The cases exhibited higher frequencies of nulliparity, family history of breast or ovarian cancer, estrogen replacement therapy in postmenopausal subjects, and chronic cigarette smoking. As expected, cases and controls had similar distributions of age, race, and education.

Table 2 shows the comparative frequencies of the medications under study with odds ratios and 95% confidence intervals. Age-adjusted and multivariate-adjusted estimates are presented. A significant reduction in the risk of breast cancer was observed for daily intake of selective COX-2 inhibitors for two years or more (OR = 0.29, 95% CI = 0.14–0.59). Significant risk reductions were also observed for the intake of two or more pills per week of regular aspirin (OR = 0.49, 95% CI = 0.26–0.95), and ibuprofen or naproxen (OR = 0.37, 95% CI = 0.18–0.72). Neither acetaminophen nor baby aspirin (81 mg) had any effect on the relative risk of breast cancer.

Table 3 presents risk estimates for individual selective COX-2 inhibitors (celecoxib and rofecoxib) plus dose-response data for aspirin and ibuprofen. Daily use of either 200 mg celecoxib or 25 mg rofecoxib for at least two years produced significant risk reductions (83% and 64% respectively). The trend data for OTC compounds suggests that 325 mg aspirin or 200 mg ibuprofen produced significant risk reductions when taken at least every other day for 5 or more years.

## Discussion

This is the first observation of a significant risk reduction in human breast cancer due to intake of selective COX-2 inhibitors. Standard daily dosages of celecoxib (200 mg) or rofecoxib (25 mg) taken for two or more years were associated with a 71% reduction in breast cancer risk. Comparator NSAIDs with non-selective COX-2 activity (325 mg aspirin, 200 mg ibuprofen or 250 mg naproxen) also produced significant risk reductions, although their observed effects were not as strong as selective compounds. In contrast, neither acetaminophen nor 81 mg (baby) aspirin changed the risk of breast cancer.

In general, NSAIDs inhibit cyclooyxgenase which is the key rate-limiting enzyme of prostaglandin biosynthesis [15-17]. Molecular studies show that the inducible COX-2 gene is over-expressed in human breast cancer and that COX-2 genetic expression in cancer cells is correlated with mutagenesis, mitogenesis, angiogenesis, and deregulation of apoptosis [18-20]. Over the counter NSAIDs have consistently shown antitumor effects in animal models of carcinogenesis [21], and in recent studies, striking antitumor effects of the specific COX-2 inhibitor, celecoxib, have been observed against breast cancer [22]. In breast cancer cells, COX-2 over-expression is also associated with CYP-19 P-450_arom _genetic expression and local estrogen biosynthesis [23-25]. The current study coupled with existing preclinical and molecular evidence suggest that aberrant induction of COX-2 and up-regulation of the prostaglandin cascade play a significant role in mammary carcinogenesis, and that blockade of this process has strong potential for intervention.

Enthusiasm for the use of selective COX-2 blocking agents in the chemoprevention of breast cancer and other malignancies has been tempered by reports of adverse effects on the cardiovascular system leading to the recall of popular anti-arthritic compounds, rofecoxib (Vioxx) and valdecoxib (Bextra). However, such studies involved supra-therapeutic dosages given over long periods of time without consideration of body size or individual differences in metabolism [26].

## Conclusion

We observed a significant reduction in the risk of human breast cancer due to intake of selective COX-2 inhibitors. Chemopreventive effects against breast cancer were associated with recommended daily doses of celecoxib (median dose = 200 mg) or rofecoxib (median dose = 25 mg) for an average duration of 3.6 years. Notably, selective COX-2 inhibitors (celecoxib and rofecoxib) were only recently approved for use in 1999, and rofecoxib (Vioxx) was withdrawn from the marketplace in 2004. Nevertheless, even in the short window of exposure to these compounds, the selective COX-2 inhibitors produced a significant (71%) reduction in the risk of breast cancer, underscoring their strong potential for breast cancer chemoprevention.

## Competing interests

This research was supported in part by a grant from Pfizer, New York, NY, and grant P30 CA16058 from the National Cancer Institute, Bethesda, MD.

## Authors' contributions

REH designed and directed the study. JBD coordinated data collection and quality control, and assisted in the interpretation of results. GAA assisted in the analysis and interpretation of results.

## Pre-publication history

The pre-publication history for this paper can be accessed here:



## Figures and Tables

**Table 1:** Characteristics of breast cancer cases and controls.

**Characteristic **^*a*^	**Cases (N = 323)**	**Controls (N = 649)**
**Age (yrs)**		
**<50**	21%	22 %
**50–65**	55	56
**>65**	24	22
**Mean (SEM)**	54.2 (0.7)	53.3 (0.4)
**Race**		
**Caucasian**	91 %	89 %
**All Other**	9	11
**Education**		
**< 12 yrs**	12 %	12 %
**12 yrs**	53	55
**> 12 yrs**	31	33
**Parity**		
**Nulliparous**	6 %	4 %
**First Pregnancy <30 yrs**	83	89
**First Pregnancy >30 yrs**	11	7 (p < 0.05)
**Family History**		
**Positive**	29 %	16 %
**Negative**	71	84 (p < 0.01)
**Body Mass**		
**BMI < 22**	23 %	21 %
**BMI 22–28**	35	39
**BMI > 28**	42	40
**Mean (SEM)**	27.5 (0.9)	27.1 (0.7)
**Menopausal Status**		
**Premenopausal**	41 %	47 %
**Postmenopausal**	59	53 (p < 0.05)
**Postmenopausal ERT**	38	31 (p < 0.05)
**Smoking**		
**Never smoker**	45 %	40 %
**Ex-smoker**	38	40
**Current smoker**	27	20 (p < 0.02)
**Alcohol Intake**		
**None**	47 %	45 %
**1–2 drinks per week**	36	35
**> 2 drinks per week**	17	20

^*a *^Family History: either breast or ovarian cancer among first or second degree female relatives; ERT = Estrogen Replacement Therapy for two or more years; Body Mass Index = weight (kg)/ht^2^(m).

**Table 2:** Odds ratios with 95% confidence intervals for breast cancer and selective cyclooxygenase-2 (COX-2) inhibitors, and over the counter nonsteroidal anti-inflammatory drugs (OTC NSAIDS).

**Compound**	**Number of Cases**	**Number of Controls**	**Age Adjusted OR (95% CI)**	**Multivariate OR**^*d *^**(95% CI)**
**None/Infrequent Use**^*a*^	262	453	1.00	1.00
**COX-2 Inhibitors**^*b*^	10	52	0.31 (0.16–0.64)	0.29 (0.14–0.59)
**OTC NSAIDs**^*c*^				
**Aspirin**	15	40	0.51 (0.27–0.98)	0.49 (0.26–0.94)
**Ibuprofen/Naproxen**	11	52	0.37 (0.18–0.71)	0.37 (0.18–0.72)
**Acetaminophen**	8	16	0.92 (0.39–2.20)	1.02 (0.39–2.20)
**Baby Aspirin**	17	36	0.82 (0.40–1.40)	0.77 (0.42–1.41)

^*a *^No use of any NSAID or analgesic or infrequent use of no more than one pill per week for less than one year;

^*b *^COX-2 inhibitors include celecoxib, rofecoxib, valdecoxib, and meloxicam used daily for two years or more.

^*c *^Over the counter (OTC) NSAIDs/analgesics used at least two times per week for two years or more.

^*d *^Multivariate odds ratios are adjusted for continuous variables (age and body mass) and categorical variables (parity, menopausal status, family history, smoking, and alcohol intake).

**Table 3:** Odds ratios for breast cancer by dose, frequency, and duration of exposure to celecoxib, rofecoxib, aspirin, and ibuprofen.

**Compound**^*a*^	**Dose**	**Frequency of Use**	**Multivariate OR**^*b *^**(95% CI)**
**Celecoxib**	200 mg	Daily	0.17 (0.03–0.83)
**Rofecoxib**	25 mg	Daily	0.36 (0.14–0.91)
**Aspirin**	325 mg	2–3 weekly	1.02 (0.30–3.57)
		>3 weekly	0.39 (0.22–0.72)
			*trend (p < 0.05)*
**Ibuprofen**	200 mg	2–3 weekly	0.59 (0.21–1.67)
		>3 weekly	0.28 (0.13–0.61)
			*trend (p < 0.01)*

^*a *^Minimum duration of exposure: 2 years for celecoxib or rofecoxib, 5 years for aspirin or ibuprofen.

^*b *^Multivariate odds ratios are adjusted for continuous variables (age and body mass) and categorical variables (parity, menopausal status, family history, smoking, and alcohol intake).
